# Unmet needs on the current medical management of Cushing’s syndrome: results from a Delphi panel of Italian endocrinologists

**DOI:** 10.1007/s40618-023-02058-8

**Published:** 2023-04-19

**Authors:** R. Pivonello, C. Scaroni, B. Polistena, A. Migliore, A. Giustina

**Affiliations:** 1 Dipartimento di Medicina Clinica e Chirurgia, Sezione di Endocrinologia, Università Federico II di Napoli, Via Sergio Pansini, 5, 80131 Naples, Italy; 2 Endocrinology Unit, Department of Medicine, DIMED, Hospital-University of Padova, Padua, Italy; 3 C.R.E.A. Sanità, Rome, Italy; 4 Institute of Endocrine and Metabolic Sciences, San Raffaele Vita-Salute University and IRCCS Hospital, Milan, Italy

**Keywords:** Cushing’s syndrome, Cushing’s disease, Rare disease, Unmet need, Cortisol, Steroidogenesis inhibitors

## Abstract

**Background:**

Cushing’s syndrome (CS) is a rare clinical condition caused by excessive cortisol secretion from adrenal glands. CS is associated with increased mortality and morbidity; therefore, a prompt diagnosis and an effective therapeutic approach are strongly necessary to improve the patient’s clinical management. The first-line treatment for CS is surgery, while medical treatment has historically played a minor role. However, thanks to the availability of novel compounds, the possibility of improving hypercortisolism control using different drug combinations emerged.

**Purpose:**

No absolute recommendations are available to guide the therapeutic choice for patients with CS and, consequently, the awareness of unmet needs in CS management is growing. Although new data from clinical trials are needed to better define the most appropriate management of CS, an expert consensus approach can help define unmet needs and optimize the current CS management and treatment.

**Methods:**

Twenty-seven endocrinologists from 12 Italian regions, working among the main Italian referral centers for hospital endocrinology where they take care of CS patients, were involved in a consensus process and used the Delphi method to reach an agreement on 24 statements about managing CS patients.

**Results:**

In total, 18 statements reached a consensus. Some relevant unmet needs in the management of CS were reported, mainly related to the lack of a pharmacological treatment successful for the majority of patients.

**Conclusion:**

While acknowledging the difficulty in achieving complete disease control, a significant change in CS management requires the availability of medical treatment with improved efficacy and safety over available therapeutic options at the time of the current study.

## Introduction

Cushing’s syndrome (CS) is a rare clinical condition caused by excessive cortisol secretion from the adrenal glands [1]. CS has an incidence of 1.5/1,000,000 inhabitants per year and a prevalence of nearly 60/1,000,000 inhabitants in Europe [2]. In approximately 80% of cases, CS is a consequence of an adrenocorticotrophin (ACTH) hypersecretion (ACTH-dependent CS), generally due to an ACTH-secreting pituitary tumor (pituitary-dependent CS or Cushing’s disease [CD], 70%), and, rarely, to an ACTH-secreting, or corticotrophin-releasing hormone-secreting, extra-pituitary tumor (ectopic CS, 10%) [3]. In the remaining 20% of cases, CS is a direct consequence of autonomous cortisol overproduction by the adrenal glands (ACTH-independent CS, adrenal CS) due to unilateral or bilateral adrenal diseases [1, 4, 5].

CS is associated with increased mortality, mostly attributable to cardiovascular complications and severe infections, as well as increased morbidity. The main comorbidities associated with CS include metabolic syndrome, cardiovascular diseases, immune disorders, musculoskeletal damage, neuropsychiatric diseases, impairment of reproductive and sexual function, together with dermatological manifestations, and suppression of pituitary function [5–15]. The entire cohort of these clinical complications substantially impairs the quality of life [5, 7–11].

A prompt diagnosis and an effective multidisciplinary therapeutic approach are strongly necessary to improve clinical picture and quality of life of patients with CS [10, 12]. Treatment goals include the normalization of cortisol levels, the reversion of clinical signs and symptoms, the prevention or improvement of concomitant comorbidities, the control of tumor growth, the long-term control of the disease without recurrence, and the restoration of normal mortality [10, 12].

The first-line treatment for CS is represented by surgery, aimed at removing the responsible tumor with consequent normalization of cortisol secretion and recovery of clinical syndrome [10, 16]. However, pituitary surgery, the main treatment of CD, is not effective in at least one-third of patients due to persistence or recurrence of the disease, therefore requiring a second therapeutic approach [10, 16]. Second-line treatments strictly depend on CS etiology and may include second pituitary surgery, medical treatment, radiotherapy and/or chemotherapy, and adrenal surgery [10].

Medical treatment has historically played a minor role in CS management. However, it has been acquiring a more important role in different steps of the treatment schedule, thanks to the availability of novel compounds and the employment of drugs previously used with different indications [5, 10, 16].

Particularly, medical treatment may be advocated before surgery, as preoperative treatment, especially in patients with severe CS, to control cortisol excess and improve the clinical picture [10]. Alternatively, it may be recommended as adjuvant treatment in patients with persistent or recurrent disease, or as bridging treatment before or after pituitary radiotherapy, while awaiting its definitive effects, or, lastly, as a primary alternative treatment in case of non-visible pituitary tumors at imaging procedures, lack of indications or contraindications to surgery, or surgery refusal [10]. The spectrum of available drugs to manage CS includes three main categories: (1) pituitary-directed drugs; (2) adrenal-directed drugs or steroidogenesis inhibitors; (3) glucocorticoid receptor-directed drugs [5, 16]. The main features of available drugs are summarized in Table 1.

**Table 1:** Main features of available drugs for the management of Cushing’s syndrome and Cushing’s disease

Drug	Description	Indication	Efficacy	References
**1. Pituitary-directed drugs (pituitary level activity: ACTH inhibition, secondary cortisol secretion inhibition)**				
Pasireotide^a^	Somatostatin analog	Approved by EMA and FDA for the treatment of CD if surgery is not an option or if surgery failed or has not been curative	Phase III study showed 15–26% UFC normalization	[5, 39–49]
Cabergoline	Long-acting dopamine agonist	Off-label treatment for CD	Retrospective studies showed approximately 20–40% UFC normalization initially, but roughly 25–40% escape	[5, 50–52]
**2. Steroidogenesis inhibitors (or adrenal-directed drugs; adrenal level activity; cortisol production and steroid hormone synthesis inhibition)**				
Ketoconazole^a^	Imidazole antifungal agent and effective inhibitor of cortisol and androgens synthesis	Approved by EMA for the treatment of CS in adults and adolescents ≥ 12 years	Retrospective studies showed approximately 65% of UFC normalization initially, but 15–25% escape	[5, 10, 53–57]
Levoketoconazole	Stereoisomer of racemic ketoconazole; inhibits key cytochrome P450 enzymes involved in multiple steps of steroidogenesis	Approved by FDA for the treatment of CS in adults if surgery is not feasible or has not been curative	Phase III open-label study showed 31% UFC normalization, 42% normalization when using imputed data	[5, 47]
Metyrapone^a^	Inhibitor of the 11β-hydroxylase enzyme that catalyzes the conversion of 11-deoxycortisol to cortisol during steroid synthesis	Approved by EMA for the treatment of CS in adults	Retrospective studies showed approximately 70% UFC normalization; UFC normalization was 47% at week 12 in a prospective study	[5, 10, 27, 58–63]
Mitotane	Antineoplastic cytostatic drug reduces the production of some steroid hormones	Approved by FDA and EMA for the treatment of adrenal cancer with endogenous CS	Retrospective studies showed that disease control might be achieved in approximately 80% of cases	[5, 64–66]
Osilodrostat^a^	Inhibitor of both steroid 11β-hydroxylase, the enzyme that catalyzes the final step in the biosynthesis of endogenous cortisol, and aldosterone synthase	Approved by EMA for adults with all the forms of endogenous CS and FDA for adults with all the forms of CS not cured by pituitary surgery or in whom pituitary surgery is not appropriate	Phase III studies showed that disease control may be achieved in 77–86% of cases	[5, 67–69]
**3. Glucocorticoid receptor (GR)-directed drugs (block the activation of GR)**				
Mifepristone	Cortisol receptor blocker	Approved by FDA for the treatment of CS patients who have type 2 diabetes or glucose intolerance and have failed surgery or are not candidates for surgery. In Europe, no official approval has been granted for mifepristone as a treatment for CS	Disease control may be achieved in 38–60% of cases in terms of blood pressure and glucose metabolism control, respectively	[5, 70, 71]

*CS* Cushing’s syndrome, *CD* Cushing’s disease, *EMA* European Medicines Agency, *FDA* Food and Drug Administration, *UFC* urinary free cortisol

^a^This drug is currently reimbursed by the Italian national health system

The availability of different drugs has raised the possibility of combined treatment, using drugs acting at different levels to improve hypercortisolism control and safety profile of the single drugs [5, 16]. Nevertheless, no absolute recommendations are available to guide this therapeutic choice [17]. On this basis, the awareness of unmet needs is growing [18].

Although new data from clinical trials are needed to better define the most appropriate management of CS, an expert consensus approach, involving endocrinologists from the centers of excellence for pituitary tumors [19, 20], may help define unmet needs regarding CS and, thus, optimizing current management and treatment, mainly considering recently published guidelines and new scientific evidence [16].

To this aim, a group of Italian endocrinologists, working among the main referral centers for hospital endocrinology, was involved in a consensus process, using the Delphi method, to reach an agreement on a list of statements on the management and treatment of patients with CS concerning the Italian scenario. This manuscript presents and critically discusses the results of this consensus activity.

## Methods

Based on the literature review and clinical experience, the authors defined the topics relevant for the analysis and the related statements through a series of online meetings held during April and May 2021. The authors developed an online survey, which was submitted to 3 experts among the authors (RP, CS, and AG) for approval and then was sent to 57 Italian endocrinologists belonging to referral centers for hospital endocrinology between June and September 2021. The large sample was chosen for the first invitation to achieve the entire cohort or at least the great majority of endocrinologists and endocrinology centers which could have expertise in CS management. A cover letter was sent to invited endocrinologists to explain the project and to invite them to answer the survey in case they have experience in CS management. The invited endocrinologists did not participate in the survey development. The invited endocrinologists, who replied to the invitation with acceptance to participate at the survey, represented the Delphi panel; the panelists used a dedicated online platform, and a timeline of 21 calendar days to respond to the survey. A further 7 days were granted after a reminder e-mail complete the process.

The survey included 24 statements based on the results coming from a literature review on the safety and clinical efficacy of current CS treatments and discussion among the authors. In some cases, the 24 statements were grouped and preceded by a brief introduction to frame the context and the rationale. The drugs reimbursed by the Italian national health system at the time of survey development, including pasireotide, ketoconazole, and metyrapone, were considered in formulating the pharmacological therapy statements. Noteworthy, osilodrostat obtained the reimbursement by the Italian national health system after the study completion.

The Delphi method was used to reach a consensus on the statements (scores on a 1–9 scale, with 1 indicating full disagreement and 9 indicating full agreement). A 70% threshold was set to define consensus, according to the most recent literature, meaning that strong disagreement or agreement was reached if at least 70% of participants had assigned scores in the range 1–3 or 7–9, respectively [21].

### Statistical analysis

All data were analyzed with descriptive statistics.

## Results

In total, 27 endocrinologists with expertise in the management of CS (47% of the identified sample) working in 12 different Italian regions participated in the survey, representing the Delphi panel. Among them, 16 (59%) reported following between 5 and 10 CS patients per year, 5 (19%) between 11 and 20 patients, 4 (15%) more than 20 patients, and 2 (7%) less than 5 patients.

The Delphi process was concluded in two rounds. In the first one, where 27 (100%) endocrinologists participated, agreement was reached on 15 (62%) out of the 24 proposed statements (numbers 1.1, 1.2, 1.3, 4.1, 5.4, 5.5, 6.1, 7.1, 7.2, 8.1, 8.2, 9.1, 9.2, 9.3, 10.1; Table 2). In the second round, where 23 (85.2%) of the initial 27 endocrinologists participated, an agreement was reached on an additional 3 (12.5%) statements (numbers 2.2, 3.1, and 5.2; Table 2). Consequently, at the end of the Delphi process, 18 (75%) statements out of 24 reached the consensus (Table 2); otherwise, 6 (25%) statements did not reach the consensus (numbers 2.1, 3.2, 3.3, 4.2, 5.1, and 5.3; Table 3).

**Table 2:** Results of Delphi panel voting

Statements	Consensus score^a^ (%)		
	1–3 (disagreement)	4–6	7–9 (agreement)
**1. Transsphenoidal surgery, often considered the first-line treatment for Cushing’s disease, is resolved in 80% of cases. Success rates are high among centers of excellence and may vary according to several factors (e.g., tumor preoperative visualization, size, and location, surgeon experience). In addition, surgery is often repeated in the second line, following a first, non-resolutive intervention (30–40% of cases)**			
1.1 The surgical management of Cushing’s disease has limitations related to the patient’s characteristics (e.g., inoperability due to comorbidity), the difficulty of the surgery technique (location and small size of tumor that make it very difficult to view), or the availability of specialists at the facility	11	8	81
1.2 The short-term success of the procedure does not exclude that the patient may have a recurrence in the medium to long term; therefore, a constant and periodic follow-up is necessary	0	4	96
1.3 Bilateral adrenalectomy is considered a necessary treatment for some forms of Cushing’s syndrome and represents a decisive treatment in about 97% of cases. Nevertheless, important limitations exist, such as permanent adrenal insufficiency leading to the need for lifetime replacement therapy with corticosteroids and tumor progression (Nelson’s syndrome)	4	18	78
**2. The limitations of the surgical approach to Cushing’s syndrome imply that a significant proportion of patients are not eligible for this type of treatment or do not fully resolve the disease and require alternative treatments, such as drug therapy**			
2.2^b^ The availability of drug treatment with improved efficacy and safety characteristics could change the management of Cushing’s syndrome	0	9	91
**3. Urinary-free cortisol is currently the benchmark for controlling/monitoring Cushing’s syndrome**			
3.1^b^ Current drug therapies allow a reduction of cortisol values but do not always determine its normalization (values within the normal range) and consequently the patient’s control	0	26	74
**4. Certain drugs currently reimbursed by the Italian national health system for treating Cushing’s syndrome are not supported by sufficiently solid clinical trial evidence**			
4.1 Therapies for Cushing’s syndrome must have a documented scientific evidence base (e.g., from randomized controlled trials)	4	18	78
**5. Oral pharmacological therapies for Cushing’s syndrome currently reimbursed by the Italian national health system present different safety and efficacy profiles**			
5.2^b^ The increase in the occurrence of AEs parallel to the increase in drug dosage is very common	0	26	74
5.4 It would be desirable to be able to control the disease more quickly than current drug therapies	8	22	70
5.5 It would be desirable to be able to control the disease for a longer period than current drug therapies	7	7	86
**6. Oral pharmacological therapies currently reimbursed by the Italian national health system require administration of up to six daily doses, with a substantial number of tablets per administration**			
6.1 This can have a very significant impact on patient compliance and consequently on the adherence and effectiveness of therapy	0	26	74
**7. Certain pharmacological treatments for Cushing’s syndrome are characterized by a significant discontinuation rate (due to side effects or lack of treatment effectiveness)**			
7.1 New drug therapies should allow a significant reduction in interruption rates	0	22	78
7.2 AEs, such as hypokalemia, acne and hirsutism and hypogonadism, may occur due to certain drug therapies and may affect not only the clinical management (requirement of supportive therapies) but also the patient’s perception of the therapy effectiveness as well as the therapy adherence	0	19	81
**8. The escape phenomenon (i.e., resumption of biochemical hypercortisolism and its clinical manifestations) observed during the different therapies for Cushing’s syndrome requires continuous dose escalation with consequent risks related to increased comorbidities and side effects**			
8.1 The escape phenomenon can have an important impact on disease control and patient management	4	4	92
8.2 New drug therapies should lead to a significant reduction in escape rates	0	11	89
**9. In a proportion of patients, Cushing’s syndrome does not resolve despite various drug treatment options reimbursed by the Italian national health system and becomes an uncontrolled chronic condition**			
9.1 There is an unmet need for pharmacological treatment able to reduce both cortisol levels and symptoms and comorbidities associated with the clinical conditions as well as restore salivary cortisol rhythm and reduce concomitant therapies used to manage comorbidities	0	15	85
9.2 A drug that can maintain long-term efficacy would be necessary, allowing medium-term to long-term disease control	0	11	89
9.3 It would be desirable for the patient to remain under control even after the treatment withdrawal	4	7	89
**10. Very few Italian regions seem to have defined pathways for patients with Cushing’s syndrome in terms of identifying centers of excellence and territorial networks**			
10.1 It is useful to encourage the development of such pathways for Cushing’s syndrome	0	0	100

Statements that reached the agreement at the end of the Delphi process (*n* = 18; 75%)

^a^Consensus is reached when at least 70% of participants assigned scores in the range 1–3 (disagreement) or 7–9 (agreement)

^b^Consensus on this statement was reached in the second Delphi round

**Table 3:** Results of Delphi panel voting

Statements	Consensus score^a^ (%)		
	1–3 (disagreement)	4–6	7–9 (agreement)
**2. The limitations of the surgical approach to Cushing’s syndrome imply that a significant proportion of patients are not eligible for this type of treatment or do not fully resolve the disease and require alternative treatments, such as drug therapy**			
2.1 A standard drug therapy for treating Cushing’s syndrome has not yet been defined	4	35	61
**3. Urinary-free cortisol is currently the benchmark for controlling/monitoring Cushing’s syndrome**			
3.2 Current drug therapies allow a reduction of cortisol values in a short time	4	61	35
3.3 Current drug therapies allow the complete control of the disease (intended as normalization of metabolic parameters, blood pressure, body weight, and bone mass)	30	65	5
**4. Certain drugs currently reimbursed by the Italian national health system for treating Cushing’s syndrome are not supported by sufficiently solid clinical trial evidence**			
4.2 Therapies for Cushing’s syndrome can be based on effectiveness and safety evidence from the clinical practice	26	56	18
**5. Oral pharmacological therapies for Cushing’s syndrome currently reimbursed by the Italian national health system present different safety and efficacy profiles**			
5.1 From a clinical point of view, the safety profiles of currently reimbursed drug therapies may be considered satisfactory	0	65	35
5.3 From a clinical point of view, the efficacy profiles (such as normalization of endocrine-metabolic parameters, body weight, and bone mass) of currently reimbursed oral pharmacological monotherapies can be considered satisfactory	9	74	17

Statements for which consensus were not reached at the end of the Delphi process (*n *= 6; 25%)

^a^Consensus is reached when at least 70% of participants assigned scores in the range 1–3 (disagreement) or 7–9 (agreement)

### Statements with agreement

Results from the Delphi panel highlighted the awareness of the current limitations of the surgical management of CD (agreement 81%) and bilateral adrenalectomy used in some forms of CS (78%), as well as the need for a periodic patient follow-up due to the risk of recurrence in the medium to long term (96%). Experts agreed on the need for new drug treatments with improved efficacy and safety to change the current management of CS (91%). Indeed, it was a shared opinion that current drug therapies allow a reduction in cortisol values but do not always determine its normalization (74%); it was also widely agreed that drug therapies for CS should have documented scientific evidence based on randomized clinical trials (RCTs, 78%), unlike certain drugs belonging to the category of steroidogenesis inhibitors reimbursed in Italy at the time of the current survey study. Regarding the current oral drug therapies for CS, most of the experts observed that the increase in drug dosage is very often paralleled by the increase in adverse events (AEs) (74%) and that it would be desirable to define new pharmacological therapies able to control the disease more quickly (70%) and for a longer period (86%) than current drug therapies. Experts agreed on the significant impact of the multiple daily administration on patient compliance and, consequently, on drug therapy adherence and effectiveness (74%). Experts shared the need to define new drug therapies able to reduce interruption rates (78%) and AEs that frequently affect clinical management mainly due to the requirement for supportive care, the patient’s perception of the therapy’s effectiveness and, therefore, the therapy adherence (81%). Experts recognized the impact of the escape phenomenon on disease control and patient management (92%) and, consequently, agreed on the need to define new therapeutic strategies to reduce escape rates (89%). Lastly, experts defined the need for a pharmacological treatment able to reduce both cortisol levels, signs, symptoms, and comorbidities, restore the rhythm of salivary cortisol, and reduce concomitant therapies (85%), allowing the medium-term to long-term control of the disease (89%), as well as the patient control, even after the treatment withdrawal (89%). All the experts encouraged the definition of patients’ pathways in terms of identifying centers of excellence and territorial networks (100%) (Table 2).

#### Statements with no consensus

Experts reported no consensus, due to failure to achieve the 70% threshold, on the availability of standard drug therapy for CS treatment (61% of agreement), on the rapid response time of the current drug therapies (35% of agreement), on the achievement of complete disease control with current drug therapies (30% of disagreement), on the opinion that CS therapies can be based on evidence from the clinical practice if data from RCTs are lacking (26% of disagreement), and on the satisfaction with the safety (35% of agreement) and efficacy profiles (17% of agreement) of the pharmacological therapies reimbursed at the time of the current survey study (Table 3).

## Discussion

Nowadays, no absolute recommendations are available to guide the therapeutic management of CS patients and, consequently, the awareness of unmet needs is growing. To address these points, a group of Italian endocrinologists were involved in a consensus process using the Delphi method. A total of 27 endocrinologists, with expertise in the management of CS, from 12 out of the 20 different Italian regions, participated in this activity, thus representing the great majority of the Italian scenario. Nearly 80% of participants reported follow-up between 5 and 20 patients with CS per year, and 13% reported to follow-up more than 20 patients with CS per year, with only 7% of participants reporting to follow-up less than 5 patients. Considering that the number of patients with pituitary diseases is not large, and CS is a rare disease, these data suggest the proper selection of participants with a good grade of expertise. The results of the current study were able to highlight some relevant unmet needs, mainly related to the lack of an effective and safe pharmacological treatment successful for the majority of patients.

### Surgical approach

Literature evidence suggests that pituitary surgery is widely considered the first-line treatment in CD management, even considering that an optimal success rate is reported especially among centers of excellence and may vary according to different factors, including patients’ characteristics, and preoperative visualization, size, and location of the pituitary tumor, as well as the surgeon experience [10, 16, 22, 23]. Consistent with this evidence, Delphi panel outcomes highlighted that the CD surgical management has some limitations (81% of agreement), mainly related to patients’ characteristics (refusal of surgery, comorbidities increasing the anesthesiologic/surgical risk, non-accessibility to all patients [15]), and to surgery issues, such as invisible or small size tumors, as well as unfavorable location or extrasellar expansion of the pituitary tumor. However, a minority of the respondents (11%) did not fully agree, likely based on recent data questioning the role of pre-surgical visualization or localization of the tumor on the surgical outcome [24]. Lastly, the unavailability of neurosurgeons with adequate expertise at the facility and, the possibility of medium-term to long-term relapse, requiring a mandatory periodical follow-up, even after a successful treatment, may impact on pituitary surgery success rate (96% of agreement).

Bilateral adrenalectomy was also recognized as a necessary treatment for CS in selected cases, especially in the case of failure of the remaining therapeutic approaches (78% of agreement). However, in line with literature evidence, experts agreed on the important limitations of this approach, such as permanent adrenal insufficiency leading to the need for lifelong glucocorticoid replacement therapy, with a high risk of developing acute adrenal crisis and corticotroph tumor progression (Nelson’s syndrome), particularly in the case of an evident pituitary tumor (78% of agreement) [10, 25].

Considering the current limitations of surgical approaches, implying that a significant proportion of patients are not eligible for these types of treatments or do not fully resolve the disease, most experts agreed that the availability of new drugs with improved efficacy and safety characteristics could change the CS management in a significant percentage of patients requiring alternative treatments to surgery (91% of agreement).

### Pharmacological management

Results of the Delphi panel reported a wide agreement on relevant unmet medical needs in the pharmacological management of CS.

The measure of urinary free cortisol is currently the benchmark for controlling and monitoring CS. However, experts agreed that current pharmacological therapies for treating CS allow a reduction of cortisol values but do not always determine its normalization and, consequently, the patient control (74% of agreement). Moreover, experts agreed that most of the drugs reimbursed by the Italian national health system at the time of the current survey study, particularly the steroidogenesis inhibitors, do not have documented scientific evidence base due to the lack of randomized controlled trials (78% of agreement). Therefore, experts agreed on the need for new drug therapies with improved efficacy and safety profiles, especially drugs associated with rapid (70% of agreement) and prolonged (86% of agreement) disease control.

Although an acceptable overall safety profile for the different drugs approved for use in CD was reported [26–28], experts also agreed on the relevance of the issues related to treatment compliance, safety, and escape. Current oral pharmacological therapies require administration of multiple daily doses, with a significant impact on patient compliance and consequently on adherence and real effectiveness of therapy (74% of agreement). Moreover, certain drugs are characterized by a significant discontinuation rate due to AEs, which often increase in parallel with the increase in drug dosage (74% of agreement). Moreover, AEs may strongly influence clinical management, with the request of add-on supportive therapies, the patient’s perception of the treatment effectiveness, as well as therapy adherence (81% of agreement). Therefore, administration modalities should be improved, and AEs reduced, to significantly decrease interruption rates (78% of agreement). Finally, treatment escape has been recognized to impact disease control and patient management (92% of agreement). Therefore, its rate should be significantly reduced within new pharmacological therapies (89% of agreement).

To optimize the overall management of CS, new pharmacological therapies should be able to control not only cortisol levels but also clinical picture, in terms of signs, symptoms, and comorbidities, as well as potentially restore the circadian rhythm of cortisol and reduce concomitant therapies used to manage comorbidities (85% of agreement).

Due to the limitations of available drugs, CS medical treatment was considered a salvage therapy in most cases or a short-term bridge therapy. Nonetheless, experts agreed that new pharmacological therapies should safely enable disease control for the medium to long term (89% of agreement).

Lastly, in line with the most recent literature, the Delphi panel also unanimously agreed (100%) on the necessity to establish centers of excellence and territorial networks to optimize the management of CS patients based on multidisciplinary and individualized approaches, encouraging the development of specific “pathways” [19, 20]. For instance, in case of undetectable pituitary tumor or microadenoma, bilateral inferior petrosal sinus sampling (BIPSS) is suggested by current guidelines for CD diagnosis [16]. However, a recent study reported that in patients with pituitary microadenoma or non-visible tumor, a concordant positive response to non-invasive tests seems sufficient to diagnose CD, irrespective of MRI finding, and that the result of surgery is not influenced by the performance of BIPSS [24]. This finding is of relevance in particular for centers where BIPSS is not feasible.

### Areas of debate

About one-third (35%) of the experts were uncertain about the existence of defined standard medical therapy for those patients not eligible for surgery or did not fully resolve after surgery, requiring alternative treatments to surgery. Notably, the most recent guidelines support using adrenal steroidogenesis inhibitors for rapid cortisol normalization among available therapeutic options [16]. This may reflect unresolved issues concerning their tolerability and efficacy, depending on individual patients’ characteristics as well as local availability and costs of each drug, which may lead to the choice of different adrenal steroidogenesis inhibitors to tailor therapy, that is recommended as the best therapeutic approach [16, 28]. For instance, although no rigorous data support the use of preoperative medical therapy, the use of adrenal steroidogenesis inhibitors can be considered if surgery is delayed [16, 19], at least in patients with severe disease who have potentially life-threatening complications. In addition, preoperative medical therapy could protect against proinflammatory and procoagulant states in the peri-operative phase [29–31]. On the other hand, adrenal steroidogenesis inhibitors do not directly target the pituitary tumor in the case of CD; therefore, they may not restore hypothalamic–pituitary–adrenal axis circadian rhythm [28], and their use may increase the risk of pituitary tumor enlargement [32]. Notably, a new meta-analysis highlighted the role of the somatostatin receptor ligand pasireotide, which is considered an alternative drug to steroidogenesis inhibitors, in the reduction of size in corticotroph pituitary tumor, strengthening the role of this treatment particularly in patients with tumor with an invasive behavior, progressive growth and/or extrasellar extension, with a low likelihood of surgical gross-total removal, or with large postoperative residual tissue [33]. Considering that up to 40% of CD patients achieved significant tumor shrinkage, this suggests a novel use of this medical treatment.

A lack of consensus was reported regarding the timing of the response to drug treatments. In detail, moderate agreement on the possibility of achieving short-time cortisol reduction with the current drug therapies was reached only by 61% of experts. This can be related to the absence of a reference time frame in the statement formulation. Indeed, treatment response can depend on the type of patient, gender, drug doses, disease duration, general state of health, or clinical history. Interestingly, this view can be changed by collecting additional real-life data, particularly for the new therapeutic options.

About one-third of respondents (30%) disagreed with the statement about the ability of current drug therapies to achieve complete disease control, intended as normalization of metabolic parameters, blood pressure, body weight, and bone mass; however, most respondents (65%) expressed moderate agreement with this statement, implying that perhaps complete control is obtained on only some of the reported parameters. Similarly, 74% of respondents were uncertain about the efficacy profiles of drugs reimbursed by the Italian national health system at the time of the survey study; about 9% were unsatisfied. The distribution of scores is probably affected, as above, by the presence of different outcome parameters to be considered in the global evaluation of the treatment outcome.

Although most of the experts agreed that therapies for CS must have documented scientific evidence based (e.g., randomized clinical trials; 78% of agreement), evidence from the clinical practice represented a matter of debate. A percentage of respondents (26%) appeared to disagree strongly; however, 56% considered this compromise acceptable. This suggests that, despite the peculiarities of this rare clinical condition, the robustness of the evidence should rely on both controlled trials and real-life data as well reflected by approval of all discussed drugs by the major drug agencies worldwide, including the FDA and EMA. Lastly, although it was recognized that an increase in AEs is very common with increasing drug dosages, most respondents (65%) were uncertain about their satisfaction with the efficacy and safety profile of currently reimbursed drug therapies (74% and 65%, respectively). However, only 35% of participants reported a high degree of satisfaction with the safety of the available therapeutic options.

### Study limitations

A limitation of the current study relies on the fact that the survey was addressed to endocrinologists belonging to the main Italian referral centers for hospital endocrinology but only 27 of the 57 invited endocrinologists (47% of the identified sample) lastly participated at the survey. However, the relatively low participation to the survey did not impact the representativeness of the responses collected, since these centers are equally distributed throughout Italy and 60% of the Italian regions are represented, taking care of a different number of patients yearly, expression of the different realities among different referral centers. Notably, the relatively low participation to the survey may have been highly impacted by the emergency time due to the COVID-19 pandemic [34, 35]. It cannot be excluded that, in some instances, collected responses could have been influenced by possible misunderstandings of the questions, since no queries were issued to centers providing answers discordant from the majority of the respondents. However, in the opinion of the authors, discrepant results more likely depend on grey areas and conflicting published results and even recommendations in several areas of CS management.

## Conclusion

The results of this consensus activity reported some relevant unmet needs in managing CS, mainly related to the lack of an effective and safe pharmacological treatment successful for the majority of patients. Up to date, the superiority of one drug over another could not be determined due to the lack of head-to-head controlled studies; moreover, the availability of a pharmacological treatment able to control hypercortisolism in the totality of CS patients and able to maintain the therapeutic effects during time is still an unmet clinical need [36]. Some pharmacological therapies in CS, such as ketoconazole and metyrapone, were approved in Europe based on small retrospective observational studies with mainly an empirical/pragmatic approach regarding dose finding [36]. Otherwise, the novel steroidogenesis inhibitor osilodrostat, recently achieving the reimbursement by the Italian health system, has been tested in prospective, randomized, phase III studies (LINC 3 and LINC 4), which showed significant and sustained normalization of cortisol levels vs placebo in CD naive patients or after unsuccessful pituitary surgery or irradiation (86% vs 29% and 77% vs 8% of patients in LINC 3 and LINC 4 study, respectively), associated with improved signs and comorbidities of CD and favorable safety and tolerability profiles [37, 38]. Considering the activity of osilodrostat, it appears to have the highest efficacy among the steroidogenesis inhibitors, followed by metyrapone and ketoconazole [5, 37], and, according to the authors’ opinion, it seems to have the appropriate profile to fill the gap in medical treatment.

In conclusion, while acknowledging the complexity of defining standard drug therapy for CS and the difficulty of achieving complete control of this clinical condition, a significant change in the CS therapeutic approach seems possible, considering the newly available treatment options. Moreover, the establishment of centers of excellence and territorial networks must be encouraged to optimize the management of CS patients based on multidisciplinary and individualized approaches.

## Data Availability

Materials described in the manuscript will be available to any researcher wishing to use them for non-commercial purposes, without breaching participant confidentiality. All datasets on which the conclusions of the paper rely will be available to readers. Datasets are presented in the main manuscript.
